# Graft Arteritis Due to *Candida* Spp. After Kidney Transplant: A Systematic Review of Individual Cases

**DOI:** 10.1093/ofid/ofaf554

**Published:** 2025-09-02

**Authors:** Praveen Kumar Tirlangi, Kiran Pothumarthy Venkata Swathi, Ravindra Attur Prabhu, Gagandeep Singh, Aleksandra Barac, Martin Peter Grobusch, Nitin Gupta

**Affiliations:** Department of Infectious Disease, Kasturba Medical College, Manipal, Manipal Academy of Higher Education, Manipal, India; Department of Infectious Disease, Kasturba Medical College, Manipal, Manipal Academy of Higher Education, Manipal, India; Department of Nephrology, Kasturba Medical College, Manipal, Manipal Academy of Higher Education, Manipal, India; Department of Microbiology, All India Institute of Medical Sciences, New Delhi, India; Clinic for Infectious and Tropical Diseases, University Clinical Center of Serbia, Faculty of Medicine, University of Belgrade, Belgrade, Serbia; Center of Tropical Medicine and Travel Medicine, Department of Infectious Diseases, Amsterdam University Medical Centers, University of Amsterdam, Amsterdam Public Health—Global Health, Amsterdam Infection & Immunity, Amsterdam, The Netherlands; Masanga Medical Research Unit (MMRU), Masanga, Sierra Leone; Centre de Recherches Médicales en Lambaréné, Lambaréné, Gabon; Institut für Tropenmedizin und Deutsches Zentrum für Infektiologie (DZIF), Universität Tübingen, Tübingen, Germany; Institute of Molecular Medicine and Infectious Diseases, University of Cape Town, Cape Town, South Africa; Department of Infectious Disease, Kasturba Medical College, Manipal, Manipal Academy of Higher Education, Manipal, India

**Keywords:** arteritis, candida, kidney, transplant

## Abstract

**Background:**

Graft arteritis due to *Candida* species (GAC) is a rare but life-threatening complication in kidney transplant recipients. This systematic review examines its clinical profile and outcomes.

**Methods:**

A PRISMA-compliant systematic review was conducted. Cases of GAC in kidney transplant recipients were identified and analyzed for clinical characteristics and outcomes.

**Results:**

Sixty-one patients from 31 studies were included. Median time to infection was 30 days (IQR: 12–60 days) post-transplant. Common symptoms included fever (41.5%) and abdominal pain (33.9%). Aneurysmal rupture occurred in 49.1%, often linked to early presentation. Surgical intervention, mainly external iliac artery ligation, was required in most cases. Mortality was 22.9% and was associated with candidemia and a significantly shorter interval between transplantation and clinical presentation.

**Conclusions:**

GAC typically occurs early after kidney transplantation, with high rates of aneurysmal rupture and mortality.

Graft arteritis due to *Candida* spp (GAC) is a rare but potentially fatal type of invasive fungal infection in kidney transplant recipients [1]. Fungal invasion of the arterial wall causes inflammation, vascular weakening, aneurysm formation, and possible rupture [2]. It usually happens in the early post-transplant phase [3]. GAC has high mortality and may require a combined medical and surgical approach [4]. Due to immunosuppressive treatment, surgical procedures, and the frequent use of indwelling catheters, kidney transplant recipients are naturally susceptible to fungal infections [5]. Numerous virulence factors, such as phospholipase activity and biofilm formation, are present in *Candida* species, especially *Candida albicans*, which promote vascular invasion and endothelial damage, ultimately leading to arteritis [2]. GAC can be caused by infections from the donor, perioperative contamination, or the translocation of the recipient's natural fungal flora [1, 3, 6]. Extended stays in the donor intensive care unit, multiorgan donation, graft contamination during retrieval, and contaminated preservation fluids are risk factors for transmission [4].

Because of its nonspecific presentation and overlap with other post-transplant complications like bacterial infections, rejection, or vascular thrombosis, the clinical diagnosis of GAC is still challenging. Because blood cultures frequently have low sensitivity, they might not identify the infection until vascular complications arise [1, 6, 7]. Although aneurysmal changes or vessel abnormalities suggestive of arteritis may be detected by imaging modalities, intraoperative findings and histopathological analysis of infected vascular tissue are usually necessary for a definitive diagnosis. A multimodal approach is necessary for management, including surgical debridement, aggressive systemic antifungal therapy, and graft removal [4]. High rates of mortality, graft loss, and rebleeding persist despite these interventions [7–11]. The clinical spectrum of this condition needs to be better understood because there is a dearth of consolidated data on it. To assess clinical features, risk factors, microbiological results, treatment approaches, and patient outcomes, this systematic review (SR) aimed to summarize individual case reports and case series to thoroughly examine GAC in kidney transplant recipients.

## METHODOLOGY

### Registration

The study adhered to the Preferred Reporting Items for Systematic Reviews and Meta-Analyses (PRISMA) guidelines (PROSPERO registration ID: CRD420251001072) [12]. The PRISMA checklist is attached as a Supplementary supplement.

### Search Strategy and Study Selection

A comprehensive literature search was conducted across three major databases: PubMed, Embase, and Web of Science. The search strategy incorporated the following keywords: (candid* OR yeast OR fungal) AND (graft OR transplant OR kidney OR renal) AND (arteritis) to identify relevant studies. All articles published between 1 January 1900, and 1 January 2025, in all languages were included in the review. To ensure comprehensive coverage, a post-hoc analysis was performed to identify articles potentially missed by the initial search strategy or those published subsequently. This additional search, conducted on 20 July 2025, employed an expanded set of keywords, notably including terms such as “endovascular infection,” “mycotic aneurysm,” and “pseudoaneurysm”, which were not explicitly part of the initial search string.

The screening process was conducted in two phases using the Rayyan AI software. In the first phase, all retrieved articles were screened by title and abstract to identify potentially relevant studies. Two independent reviewers (T. P. K. and P. V. S. K.) conducted this screening, and a third reviewer (N. G.) resolved discrepancies. In the second phase, full-text articles of the shortlisted studies were retrieved and assessed for eligibility. The same two reviewers independently reviewed the full texts. Citations of the included articles were thoroughly searched to search for additional articles.

GAC was defined as inflammation leading to a pseudoaneurysm or rupture of a transplanted renal artery or its anastomosis with the external iliac artery, definitively diagnosed by positive intraoperative culture, histopathology, or blood cultures for *Candida spp*. We only included studies that provided individual case details on GAC's clinical profiles and outcomes in kidney transplant recipients, specifically those where the diagnosis occurred within the first year post-transplant. Studies involving other transplanted organs alongside the kidney (eg, liver, pancreas) were excluded. Studies with inadequate clinical details, especially concerning the outcomes, were excluded. Cases where organisms other than *Candida* spp were also cultured from the intraoperative samples were also excluded. Conference abstracts, narrative reviews, systematic reviews, and viewpoints were excluded. Any disagreements in the full-text screening phase were again resolved through discussion or consultation with a third reviewer.

### Quality Assessment and Critical Appraisal

All included cases were critically assessed using the Joanna Briggs Institute appraisal tool for case reports. The quality assessment focused on the completeness of patient details, diagnostic accuracy, therapeutic interventions, and outcome documentation [13].

### Data Extraction

Two independent reviewers extracted data using a predefined data collection form. Extracted variables included patient demographics, transplant characteristics, clinical presentation, microbiological findings, antifungal prophylaxis, treatment strategies, and clinical outcomes. Any discrepancies in data extraction were resolved through discussion or consultation with a third reviewer.

### Statistical Analysis

Extracted data were analyzed descriptively, with percentages calculated for variables where numerator and denominator data were available. Continuous variables were presented as means with standard deviations, while categorical variables were reported as frequencies and percentages. Comparative analyses were conducted for key outcomes such as aneurysmal rupture, rebleeding risk, and mortality. The impact of different surgical strategies on rebleeding rates was assessed. Differences between groups were evaluated using t-tests for continuous variables and chi-square or Fisher's exact tests for categorical variables. Statistical significance was set at *P* < .05.

## RESULTS

### Screening Results

**Figure 1.**
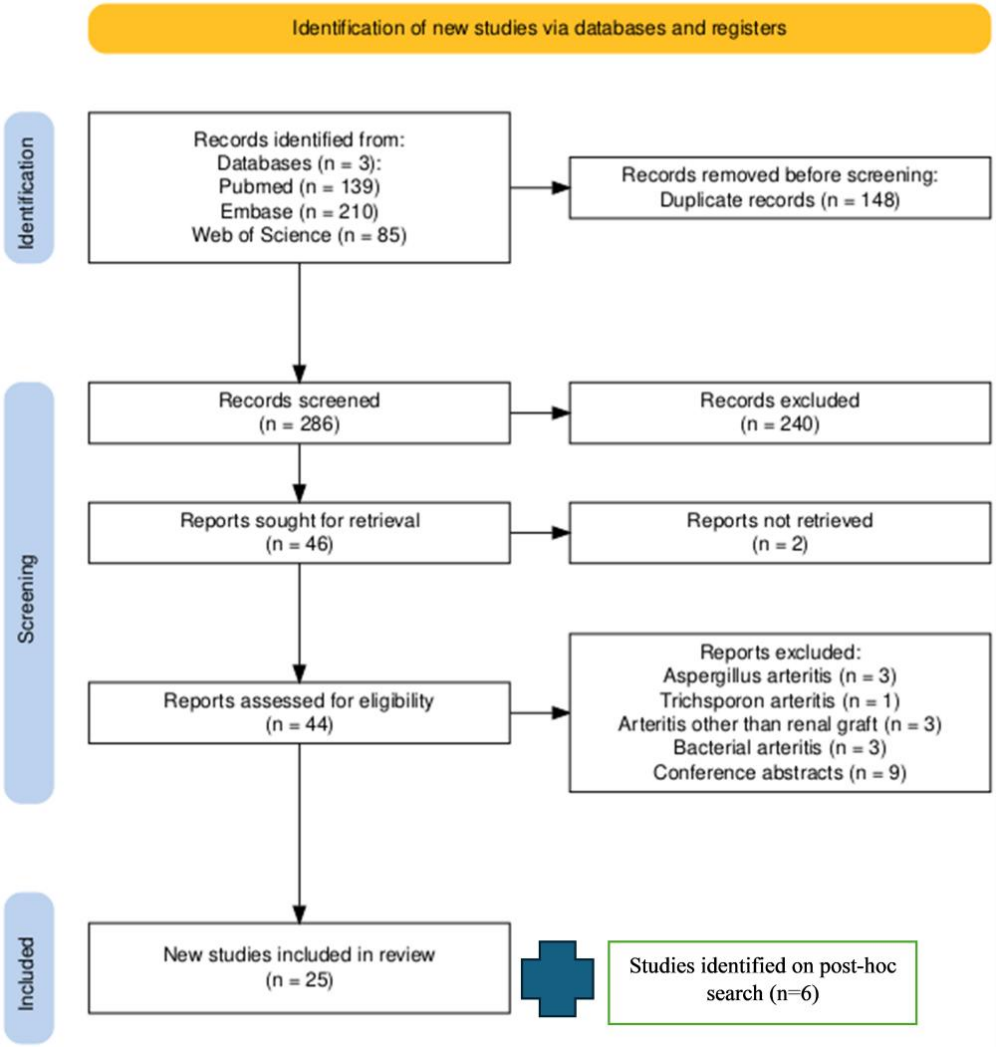
PRISMA Flow diagram for study selection on studies with individual details available for patients with *Candida arteritis*.

The initial search retrieved 434 articles, 139 from PubMed, 210 from Embase, and 85 from Web of Science. After removing 148 duplicate records, 286 unique studies remained for title and abstract screening. Based on relevance to the research question, 44 articles were selected for full-text review. During this stage, nine studies were excluded as conference abstracts, three due to arteritis other than kidney graft artery involvement, and seven because they involved organisms other than *Candida* spp. After applying these exclusion criteria, 25 studies (with 52 cases) were included in the final analysis (Figure 1). Six additional articles (with nine cases) were included in a post-hoc search, making a total of 31 articles with 61 cases [1, 3, 6–8, 10, 11, 14–37]. All articles were published between 1998 and 2025 [1, 3, 6–8, 10, 11, 14–37]. Most of the studies were published in the United States (n = 13). Supplementary Table 1 provides a complete overview of the important original data extracted from the studies included.

### Demographic Features

Among the 47 patients with available gender data, 33 (70.2%) were male. The mean age was 42.6 ± 14.8 years. Comorbidity data were available for 35 patients, among whom diabetes mellitus was reported in six (17.1%) and hypertension in eight (22.8%) patients.

### Details of Transplantation

Among the 47 patients with available donor details, 44 (93.6%) received kidneys from deceased donors. Induction chemotherapy details were available for 23 patients, with anti-thymocyte globulin (ATG) used in 11 (47.8%) and IL-2 blockers in 11 (47.8%). No induction chemotherapy was given for one patient. Maintenance immunosuppressive therapy included steroids in 35/36 patients (97.2%), calcineurin inhibitors (CNI), such as tacrolimus, in 33/36 (91.6%) patients, mammalian target of rapamycin (mTOR) inhibitors such as sirolimus in two patients (5.5%), and antimetabolites, such as mycophenolate mofetil in all 36 (100%) patients. Immediate postoperative complications included Gram-negative infections in 4/43 (9.3%) patients. Delayed graft function occurred in 10/42 (23.8%) patients. Acute rejection was reported in 4/43 (9.3%) patients.

### Clinical Features of Candida Arteritis at Presentation

GAC developed at a median of 30 days post-transplantation (IQR:12–60 days). Common presenting symptoms included fever in 22/53 (41.5%) patients, abdominal pain in 18/53 (33.9%) patients, and hematuria in 2/53 (3.8%) patients. Aneurysmal vessel rupture (30/61, 49.1%) was the main reported complication.

Among the 31 patients who did not present with aneurysmal rupture, aneurysm size with specific measurements was available in 11 cases, ranging from 1.4 to 6 cm. Aneurysmal thrombosis occurred in 5/60 patients (8.3%).

### Microbiology and Prophylaxis

*Candida spp* associated with GAC was identified either on the histopathology or culture of the intraoperative tissue or blood culture. Concurrent candidemia was present in 7/34 (20.6%) patients. *Candida albicans* (53/61, 86.9%) was the most common species associated with GAC. *C. glabrata* (along with *C. albicans*) and *C. dubliniensis* were identified in one patient each. No species could be identified in seven patients.

Preservative fluid cultures also revealed *Candida* spp. in 22/34 (64.7%). Bacterial growth in the preservative fluid culture was noted in 7/34 (20.6%) patients. Prophylaxis details were available for 31 patients; 8/31 (25.8%) had received prophylactic treatment.

### Management

Azoles were the most commonly used treatment, administered to 40/54 (74.1%) patients, followed by polyenes (20/54, 37%) and echinocandins (14/54, 25.9%). Surgical details were available for 49 patients (direct vascular repair (*n* = 14, 28.6%), vascular graft (*n* = 13, 26.5%), external iliac artery (EIA) ligation with femorofemoral bypass (*n* = 22, 44.8%).

### Outcomes and Risk Factors for Poor Outcomes

Graft loss was the most common adverse outcome in 53/61 (86.8%) cases. Rebleeding was reported in 18/38 (47.3%) patients. In the analysis of rebleeding risk based on the type of surgery for *Candida arteritis*, rebleeding was most frequent among patients who underwent direct repair (11/14, 78.8%). In comparison, rebleeding occurred less commonly in patients who underwent EIA ligation (3/11, 27.2%) and in patients who received a vascular graft (4/13, 30.8%). The rebleeding risk between the types of surgeries was statistically significant (*P* = .013). Mortality was reported in 14/61 (22.9%) patients. Patients who died had a significantly shorter interval between transplantation and clinical presentation and were more likely to experience delayed graft dysfunction (Table 1). Candidemia was also significantly associated with mortality.

**Table 1.** Comparison of Patients With Graft Arteritis Due to Candida Spp. based on Mortality Outcomes

Variables	No Mortality (*n* = 47)	Mortality (*n* = 14)	*P-V*alue
Mean age (years)	42.8 ± 15.4	41.9 ± 12.8	.872^a^
Gender—Male	25/36 (69.4%)	8/10 (80.0%)	.446
Induction—ATG	10/17 (58.8%)	1/6 (16.7%)	.126
Induction—IL-2	6/17 (35.3%)	5/6 (83.3%)	
Median days from transplantation to presentation	34.5 (IQR: 15–90)	12 (IQR: 9–30)	.**010**^b^
Delayed graft dysfunction	5/34 (14.7%)	5/8 (62.5%)	.**004**
Fever	20/45 (44.4%)	2/8 (25.0%)	.304
Abdominal pain	13/45 (28.9%)	5/8 (62.5%)	.064
Candidemia	4/28 (14.3%)	3/6 (50.0%)	.**05**
Aneurysmal rupture	21/47 (44.7%)	9/14 (64.3%)	.222
Prophylaxis	4/21 (19.0%)	4/10 (40.0%)	.213
Azole for treatment	33/42 (78.6%)	7/12 (58.3%)	.158
Echinocandins for treatment	12/42 (28.6%)	2/12 (16.7%)	.407
Amphotericin-B for treatment	16/42 (38.1%)	4/12 (33.3%)	.763
Surgery-direct	11/43 (25.6%)	3/6 (50.0%)	.46
Surgery-external iliac artery ligation	20/43 (46.5%)	2/6 (33.3%)	
Surgery-vascular graft	12/43 (27.9%)	1/6 (16.7%)	

ATG: Anti-thymocyte globulin, IL-2: Interleukin receptor antagonist.

^a^Independent *t*-test.

^b^Mann–Whitney *U*-test.

Chi-square test used for rest. The significant *P*-value is written in bold letters.

### Anastomotic Rupture vs no Rupture

Patients with anastomotic rupture and bleeding had a significantly shorter duration from transplantation to presentation and were less likely to present with fever or abdominal pain (Table 2).

**Table 2.** Comparison of Patients With Graft Arteritis Due to Candida Spp. based on Aneurysmal Rupture

Variables	No Aneurysmal Rupture (*n* = 31)	Aneurysmal Rupture (*n* = 30)	*P V*alue
Mean age (years)	45.9 ± 15.6	39.1 ± 13.2	.118^a^
Male gender	17/24 (70.8%)	16/23 (69.6%)	.924
Induction chemotherapy—ATG	7/13 (53.8%)	4/10 (40.0%)	.462
Induction chemotherapy—IL-2	5/13 (38.5%)	6/10 (60.0%)	
Median days from transplantation to presentation	60 (41.5–90)	15 (9–26.5)	**<.001** ^b^
Fever	16/30 (53.3%)	6/23 (26.1%)	.**046**
Abdominal pain	6/30 (20.0%)	12/23 (52.2%)	.**014**
Candidemia	5/14 (35.7%)	2/20 (10.0%)	.068
Mortality	5/31 (16.1%)	9/30 (30.0%)	.142
Graft loss	25/31 (80.6%)	28/30 (93.3%)	.198

ATG: Anti-thymocyte globulin, IL-2- Interleukin receptor antagonist.

^a^Independent *t*-test.

^b^Mann–Whitney *U*-test.

Chi-square test used for the rest. The significant *P*-value is written in bold letters.

### Critical Appraisal of the Studies

The critical appraisal revealed varying levels of completeness in reporting (Supplementary Table 2). Most cases clearly described the patient's presentation (n = 53), diagnostic methods (n = 61), and follow-up (n = 61). However, the inclusion of demographic details (n = 47) and patient history (n = 28) was inconsistent, with many instances lacking these crucial elements. Additionally, 46 cases clearly described the treatment or intervention, but some reports omitted this information.

## DISCUSSION

In this SR, we analyzed all 31 studies published between 1998 and 2024, covering 61 cases of GAC. Most patients had undergone deceased donor kidney transplants, with a median infection onset of one month post-transplant. The most frequently reported symptoms were fever and abdominal pain. Aneurysmal rupture occurred in half of the cases, often presenting earlier and with a lower incidence of fever. Surgery was necessary for most patients, with external iliac artery ligation performed in 45% of cases. Direct vascular repair was associated with a higher risk of rebleeding. The overall prognosis was poor, with a mortality rate of 23%.

This SR's results highlight several important clinical characteristics of kidney transplant recipients' GAC. GAC appears to present clinically in two different ways: (1) early in the post-transplant period, when an anastomotic site rupture is usually present and frequently occurs without fever; and (2) the subacute form, where an aneurysm is discovered by chance, with or without fever. The severe immunosuppression during the perioperative phase may account for the lack of fever in early cases. Acute rejection, reperfusion injury, bacterial infections, delayed graft function, or mechanical vascular abnormalities are some post-transplant complications that GAC can closely resemble during this period [4]. Even when there are no aneurysmal findings on surveillance kidney Doppler imaging, anastomotic rupture with hemorrhage may occasionally be the initial sign of graft arteritis [38].

One known risk factor for graft failure and patient death is delayed graft function (DGF), defined as the requirement for dialysis during the first week after transplantation [39]. Our review found that delayed graft function is associated with a higher mortality risk in patients with GAC. DGF is often a result of ischemia-reperfusion injury, which compromises endothelial integrity and triggers local inflammatory responses, though this association may be coincidental [40]. GAC may develop due to such vascular damage, making it easier for fungi to invade and colonize the vessel wall.

Most patients had undergone deceased donor kidney transplants, with a median infection onset of one month post-transplant. Multiorgan donation increases the risk of contamination during organ retrieval, especially in cases of inadvertent bowel injury during abdominal organ retrieval. An elevated risk of graft contamination is linked to non-heart-beating donors who may be susceptible to gut translocation, as well as donors who have had extended stays in intensive care units (ICUs) and extensive antibiotic exposure, which may encourage overgrowth of *Candida* spp. [4]. Culturing preservation fluid may facilitate prompt clinical decision-making and help detect fungal contamination early. In addition to receiving immediate antifungal prophylactic treatment, patients with positive preservation fluid cultures should be closely monitored clinically and radiologically for any early indications of graft arteritis. However, the low sensitivity and frequent delays in fungal growth detection of preservation fluid cultures limit their diagnostic utility. Bacterial overgrowth can further obscure the identification of *Candida* spp, explaining positive gram-negative preservative fluid cultures in patients with graft arteritis [41]. Rapid molecular techniques, such as fungal polymerase chain reaction assay or next-generation sequencing, promise to improve early diagnosis and timely antifungal therapy initiation [42, 43].

Surgical intervention remains the cornerstone of management, with graftectomy, external iliac artery ligation, and femorofemoral bypass being the preferred approach [14, 23]. Direct vascular repair following aneurysmal resection is associated with a higher risk of rebleeding [6, 7, 9–11, 14, 25–27]. Graft preservation was attempted in select cases; however, the ideal criteria for such conservative management remain unclear [3, 7, 14, 21, 23]. Outcomes highlight the poor prognosis of GAC, with high mortality, especially in cases with aneurysmal rupture or delayed diagnosis. Graft loss was also common, stressing the need for early prophylactic antifungals, recognition, and treatment.

Beyond the absence of control groups and inherent biases of case reports, our review has additional limitations, including the lack of data on contamination during organ retrieval, antifungal prophylaxis initiation timing, immunosuppression modifications, and the rationale for choosing direct vascular repair over EIA ligation with femoral bypass in certain patients. A significant limitation of this systematic review is the inherent risk of publication bias, which is difficult to quantify when analyzing case reports. Milder disease presentations, such as cases without aneurysms or those resolved with conservative therapy, are likely underreported, potentially skewing the evidence toward more severe outcomes. Another key limitation is the small sample size, which reduces the statistical power of the analysis.

## CONCLUSIONS

GAC is a rare but devastating complication in kidney transplant recipients, characterized by nonspecific symptoms, diagnostic delays, and poor outcomes. Improving prognosis requires heightened clinical vigilance, yet the lack of standardized protocols and centralized data impedes optimal management. Future research should prioritize determining the need for peri-transplant antifungal prophylaxis, especially in high-risk patients.

## Supplementary Material

ofaf554_Supplementary_Data

## Supplementary Data

Supplementary materials are available at *Open Forum Infectious Diseases* online. Consisting of data provided by the authors to benefit the reader, the posted materials are not copyedited and are the sole responsibility of the authors, so questions or comments should be addressed to the corresponding author.
