# Supplementary material for: Graft Arteritis Due to Candida Spp. After Kidney Transplant: A Systematic Review of Individual Cases
Source: Open Forum Infect Dis. 2025 Sep 2;12(10):ofaf554. doi: 10.1093/ofid/ofaf554 (PMC12526878; doi:10.1093/ofid/ofaf554)
Supplement: ofaf554_Supplementary_Data [file ofaf554_supplementary_data.zip › Supplementary Tables.docx]

Supplementary Table 1: Characteristics of the included cases with graft arteritis due to *Candida* spp

| Sn | **Author** | **Age** | **Gender** | **Donor** | **Time from transplant (days)** | **Fever** | **Abdominal pain** | **Candidaemia** | **Aneurysmal rupture** | **Surgery** | **Rebleed** | **Moratlity** |
| --- | --- | --- | --- | --- | --- | --- | --- | --- | --- | --- | --- | --- |
| 1 | Bindi et al 2020 [1] | 18 | Male | Deceased | 70 | No | No | NA | No | Direct | No | No |
| 2 | Lazareth et al 2017 [2] | 40 | Male | Deceased | 47 | No | No | NA | No | Vascular graft | No | No |
| 3 | Ministro et al 2017 [3] | 54 | Male | Deceased | 8 | No | No | No | Yes | Direct | Yes | No |
| 4 | Ministro et al 2017 [3] | 48 | Female | Deceased | 150 | No | No | NA | No | Vascular graft | No | No |
| 5 | Muthusamy 2015 [4] | NA | NA | Deceased | 90 | No | No | NA | No | EIA ligation | No | No |
| 6 | Patrono et al 2015 [5] | 31 | Male | Deceased | 12 | No | Yes | Yes | Yes | Vascular graft | No | No |
| 7 | Guy et al 2013 [6] | 62 | Male | Live | 90 | No | No | No | No | Vascular graft | Yes | No |
| 8 | Guy et al 2013 [6] | 62 | Male | Live | 90 | No | No | No | No | EIA ligation | No | No |
| 9 | Kountidou et al 2012 [7] | 47 | Female | NA | 90 | No | No | No | No | Vascular graft | No | No |
| 10 | Taksin et 2008 [8] | 58 | Female | Deceased | 15 | Yes | Yes | No | No | Vascular graft | No | No |
| 11 | Osmon et al 2008 [9] | 15 | Male | Deceased | 36 | Yes | No | NA | No | Direct | Yes | No |
| 12 | Mai et al 2006 [10] | 30 | Male | Deceased | 9 | No | Yes | No | Yes | NA | NA | Yes |
| 13 | Mai et al 2006 [10] | 26 | Female | Deceased | 30 | Yes | No | No | Yes | EIA ligation | No | No |
| 14 | Mai et al 2006 [10] | 53 | Male | Deceased | 16 | No | Yes | No | Yes | NA | NA | Yes |
| 15 | Mai et al 2006 [10] | 26 | Female | Deceased | 17 | Yes | No | No | Yes | EIA ligation | No | No |
| 16 | Baccarani et al 2003 [11] | 35 | Male | Deceased | 28 | No | No | No | Yes | Vascular graft | Yes | No |
| 17 | Baccarani et al 2003 [11] | 24 | Male | Deceased | 18 | No | No | No | Yes | Direct | Yes | No |
| 18 | Baccarani et al 2003 [11] | 58 | Male | Deceased | 28 | No | No | No | Yes | EIA ligation | Yes | No |
| 19 | Battaglia et al 1999 [12] | 34 | Male | Deceased | 17 | No | Yes | No | Yes | Vascular graft | Yes | No |
| 20 | Battaglia et al 1999 [12] | 43 | Male | Deceased | 90 | Yes | Yes | No | No | Vascular graft | No | No |
| 21 | Potti et al 1998 [13] | 60 | Male | Deceased | 42 | No | Yes | No | Yes | Direct | Yes | No |
| 22 | Horn et al 2024 [14] | 63 | Male | Deceased | 60 | No | Yes | No | No | EIA ligation | No | No |
| 23 | Albano et al 2008 [15] | NA | NA | Deceased | 25 | No | No | NA | Yes | EIA ligation | NA | No |
| 24 | Albano et al 2008 [15] | NA | NA | NA | 30 | NA | NA | NA | Yes | EIA ligation | NA | Yes |
| 25 | Albano et al 2008 [15] | NA | NA | NA | 50 | Yes | No | NA | No | EIA ligation | NA | No |
| 26 | Albano et al 2008 [15] | NA | NA | NA | 94 | Yes | No | NA | No | EIA ligation | NA | No |
| 27 | Albano et al 2008 [15] | NA | NA | NA | 115 | Yes | No | NA | No | EIA ligation | NA | No |
| 28 | Albano et al 2008 [15] | NA | NA | NA | 33 | NA | NA | NA | Yes | EIA ligation | NA | No |
| 29 | Albano et al 2008 [15] | NA | NA | NA | 50 | Yes | No | NA | No | EIA ligation | NA | No |
| 30 | Albano et al 2008 [15] | NA | NA | NA | 9 | NA | NA | NA | Yes | EIA ligation | NA | No |
| 31 | Albano et al 2008 [15] | NA | NA | NA | 11 | Yes | No | NA | No | EIA ligation | NA | No |
| 32 | Albano et al 2008 [15] | NA | NA | NA | 8 | NA | NA | NA | Yes | NA | NA | Yes |
| 33 | Albano et al 2008 [15] | NA | NA | NA | 9 | NA | NA | NA | Yes | NA | NA | Yes |
| 34 | Albano et al 2008 [15] | NA | NA | NA | 13 | NA | NA | NA | Yes | NA | NA | Yes |
| 35 | Albano et al 2008 [15] | NA | NA | NA | 3 | Yes | No | NA | No | EIA ligation | NA | No |
| 36 | Maio 2004 [16] | 36 | Male | Deceased | 60 | Yes | No | NA | No | EIA ligation | Yes | No |
| 37 | Toussant et al 2004 [17] | 46 | Female | Deceased | 26 | No | Yes | No | Yes | Direct | Yes | No |
| 38 | Slizien et al 2014 [18] | 44 | Male | Deceased | 10 | No | Yes | Yes | Yes | Direct | No | Yes |
| 39 | Slizien et al 2014 [18] | 45 | Female | Deceased | 12 | Yes | Yes | Yes | No | Direct | Yes | Yes |
| 40 | Bozkurt et al 2010 [19] | 31 | Male | Deceased | 12 | Yes | Yes | No | Yes | Direct | Yes | No |
| 41 | Bozkurt et al 2010 [19] | 47 | Male | Deceased | 18 | Yes | Yes | NA | Yes | NA | NA | No |
| 42 | Laouad et al 2005 [20] | 39 | Male | Deceased | 90 | Yes | No | Yes | No | NA | NA | No |
| 43 | Laouad et al 2005 [20] | 55 | Male | Deceased | 50 | Yes | No | NA | No | NA | NA | No |
| 44 | Laouad et al 2005 [20] | 35 | Male | Deceased | 90 | Yes | No | NA | No | NA | NA | No |
| 45 | Laouad et al 2005 [20] | 24 | Male | Deceased | 9 | No | No | NA | Yes | NA | NA | Yes |
| 46 | Rodrigues et al 2013 [21] | 29 | Female | Deceased | 37 | No | Yes | No | Yes | EIA ligation | No | No |
| 47 | Rodrigues et al 2013 [21] | 49 | Male | Deceased | 59 | No | No | No | No | Direct | Yes | Yes |
| 48 | Lin et al 2018 [22] | 56 | Female | Deceased | 32 | No | No | No | No | Vascular graft | No | No |
| 49 | Egbuna et al 2007[23] | 22 | Female | Deceased | 60 | Yes | Yes | NA | No | EIA ligation | No | Yes |
| 50 | Bandar et al 2017 [24] | 33 | Female | Deceased | 6 | No | No | No | Yes | Vascular graft | Yes | No |
| 51 | Rodrigues et al 2013 [21] | 58 | Female | Deceased | 6 | No | Yes | No | Yes | EIA ligation | Yes | No |
| 52 | Jacobs et al 2020 [25] | 72 | Male | Deceased | 90 | No | No | Yes | No | EIA ligation | No | No |
| 53 | Praveen et al 2025 [26] | 58 | Male | Deceased | 15 | No | No | No | Yes | Direct | Yes | 0 |
| 54 | Stern et al 2021 [27] | 44 | Male | Deceased | 9 | NA | NA | NA | Yes | NA | NA | Yes |
| 55 | Stern et al 2021 [27] | 62 | Male | Deceased |  | NA | NA | NA | No | NA | NA | Yes |
| 56 | Bracale et al 2009 [28] | 40 | Female |  | 78 | Yes | Yes | NA | No | EIA ligation | NA | No |
| 57 | Calvino et al 1999 [29] | 39 | Female | Deceased | 11 | Yes | Yes | No | Yes | Direct | Yes | No |
| 58 | Calvino et al 1999 [29] | 16 | Male | Deceased | 15 | Yes | No | No | Yes | Direct | Yes | No |
| 59 | Zavos et al 2005 [30] | 65 | Male | Deceased | 150 | No | No | Yes | No | Vascular graft | No | No |
| 60 | Zavos et al 2005 [30] | 46 | Male | Deceased | 30 | No | No | Yes | No | Vascular graft | No | Yes |
| 61 | Desai M et al 2015 [31] | 23 | Male | Living | 25 | No | No | No | No | Direct | No | No |

Supplementary Table 2: Critical appraisal of the included cases using the JBI checklist for case reports

| Sn | Authors | Demography | History | Presentation | Diagnosis | Treatment | Follow-up |
| --- | --- | --- | --- | --- | --- | --- | --- |
| 1 | Bindi et al 2020 [1] | Yes | Yes | Yes | Yes | Yes | Yes |
| 2 | Lazareth et al 2017 [2] | Yes | No | Yes | Yes | Yes | Yes |
| 3 | Ministro et al 2017 [3] | Yes | Yes | Yes | Yes | Yes | Yes |
| 4 | Ministro et al 2017 [3] | Yes | Yes | Yes | Yes | Yes | Yes |
| 5 | Muthusamy 2015 [4] | No | No | Yes | Yes | Yes | Yes |
| 6 | Patrono et al 2015 [5] | Yes | No | Yes | Yes | Yes | Yes |
| 7 | Guy et al 2013 [6] | Yes | Yes | Yes | Yes | Yes | Yes |
| 8 | Guy et al 2013 [6] | Yes | Yes | Yes | Yes | Yes | Yes |
| 9 | Kountidou et al 2012 [7] | Yes | No | Yes | Yes | Yes | Yes |
| 10 | Taksin et 2008 [8] | Yes | Yes | Yes | Yes | Yes | Yes |
| 11 | Osmon et al 2008 [9] | Yes | No | Yes | Yes | Yes | Yes |
| 12 | Mai et al 2006 [10] | Yes | Yes | Yes | Yes | No | Yes |
| 13 | Mai et al 2006 [10] | Yes | No | Yes | Yes | Yes | Yes |
| 14 | Mai et al 2006 [10] | Yes | Yes | Yes | Yes | No | Yes |
| 15 | Mai et al 2006 [10] | Yes | No | Yes | Yes | Yes | Yes |
| 16 | Baccarani et al 2003 [11] | Yes | No | Yes | Yes | Yes | Yes |
| 17 | Baccarani et al 2003 [11] | Yes | Yes | Yes | Yes | Yes | Yes |
| 18 | Baccarani et al 2003 [11] | Yes | Yes | Yes | Yes | Yes | Yes |
| 19 | Battaglia et al 1999 [12] | Yes | No | Yes | Yes | Yes | Yes |
| 20 | Battaglia et al 1999 [12] | Yes | No | Yes | Yes | Yes | Yes |
| 21 | Potti et al 1998 [13] | Yes | No | Yes | Yes | Yes | Yes |
| 22 | Horn et al 2024 [14] | Yes | No | Yes | Yes | No | Yes |
| 23 | Albano et al 2008 [15] | No | No | Yes | Yes | Yes | Yes |
| 24 | Albano et al 2008 [15] | No | No | No | Yes | Yes | Yes |
| 25 | Albano et al 2008 [15] | No | No | Yes | Yes | Yes | Yes |
| 26 | Albano et al 2008 [15] | No | No | Yes | Yes | Yes | Yes |
| 27 | Albano et al 2008 [15] | No | No | Yes | Yes | Yes | Yes |
| 28 | Albano et al 2008 [15] | No | No | No | Yes | Yes | Yes |
| 29 | Albano et al 2008 [15] | No | No | Yes | Yes | Yes | Yes |
| 30 | Albano et al 2008 [15] | No | No | No | Yes | Yes | Yes |
| 31 | Albano et al 2008 [15] | No | No | Yes | Yes | Yes | Yes |
| 32 | Albano et al 2008 [15] | No | No | No | Yes | No | Yes |
| 33 | Albano et al 2008 [15] | No | No | No | Yes | No | Yes |
| 34 | Albano et al 2008 [15] | No | No | NO | Yes | No | Yes |
| 35 | Albano et al 2008 [15] | No | No | Yes | Yes | Yes | Yes |
| 36 | Maio 2004 [16] | Yes | No | Yes | Yes | Yes | Yes |
| 37 | Toussant et al 2004 [17] | Yes | No | Yes | Yes | Yes | Yes |
| 38 | Slizien et al 2014 [18] | Yes | No | Yes | Yes | Yes | Yes |
| 39 | Slizien et al 2014 [18] | Yes | No | Yes | Yes | Yes | Yes |
| 40 | Bozkurt et al 2010 [19] | Yes | Yes | Yes | Yes | No | Yes |
| 41 | Bozkurt et al 2010 [19] | Yes | Yes | Yes | Yes | No | Yes |
| 42 | Laouad et al 2005 [20] | Yes | Yes | Yes | Yes | No | Yes |
| 43 | Laouad et al 2005 [20] | Yes | Yes | Yes | Yes | No | Yes |
| 44 | Laouad et al 2005 [20] | Yes | Yes | Yes | Yes | No | Yes |
| 45 | Laouad et al 2005 [20] | Yes | Yes | Yes | Yes | No | Yes |
| 46 | Rodrigues et al 2013 [21] | Yes | Yes | Yes | Yes | Yes | Yes |
| 47 | Rodrigues et al 2013 [21] | Yes | Yes | Yes | Yes | Yes | Yes |
| 48 | Lin et al 2018 [22] | Yes | Yes | Yes | Yes | No | Yes |
| 49 | Egbuna et al 2007[23] | Yes | No | Yes | Yes | Yes | Yes |
| 50 | Bandar et al 2017 [24] | Yes | No | Yes | Yes | Yes | Yes |
| 51 | Rodrigues et al 2013 [21] | Yes | No | Yes | Yes | Yes | Yes |
| 52 | Jacobs et al 2020 [25] | Yes | Yes | Yes | Yes | Yes | Yes |
| 53 | Praveen et al 2025 [26] | Yes | Yes | Yes | Yes | Yes | Yes |
| 54 | Stern et al 2021 [27] | Yes | Yes | No | Yes | No | Yes |
| 55 | Stern et al 2021 [27] | Yes | Yes | No | Yes | Yes | Yes |
| 56 | Bracale et al 2009 [28] | Yes | No | Yes | Yes | No | Yes |
| 57 | Calvino et al 1999 [29] | Yes | Yes | Yes | Yes | Yes | Yes |
| 58 | Calvino et al 1999 [29] | Yes | Yes | Yes | Yes | Yes | Yes |
| 59 | Zavos et al 2005 [30] | Yes | Yes | Yes | Yes | Yes | Yes |
| 60 | Zavos et al 2005 [30] | Yes | Yes | Yes | Yes | Yes | Yes |
| 61 | Desai M et al 2015 [31] | Yes | Yes | Yes | Yes | Yes | Yes |

1. Bindi M, Ferraresso M, De Simeis ML, et al. Allograft artery mycotic aneurysm after kidney transplantation: A case report and review of literature. World J Clin Cases **2020**; 8:912–921.

2. Lazareth H, Burbach M, Gosset C, et al. Renal Arterial Mycotic Aneurysm After Kidney Transplantation. Urology **2017**; 106:e7–e8.

3. Ministro A, Ferreira T, Batista L, et al. Mycotic Pseudoaneurysm After Kidney Transplantation: Two Case Reports. Transplant Proc **2017**; 49:906–912.

4. Rathnasamy Muthusamy AS, Hammer C, Somasundaram M, Friend P, Darby C. On Candida arteritis in renal transplant recipients. Transpl Infect Dis **2015**; 17:929–930.

5. Patrono D, Verhelst R, Buemi A, Darius T, Godefroid N, Mourad M. Presentation and management of mycotic pseudoaneurysm after kidney transplantation. Transpl Infect Dis **2015**; 17:129–136.

6. Guy A, Chinai N, Ashley S, Rowe P, Cunningham R, Barwell J. Candida arteritis in a pair of renal transplant recipients. Exp Clin Transplant **2013**; 11:558–561.

7. Kountidou CS, Stier K, Niehues SM, et al. Successful repair of post-transplant mycotic aneurysm of iliac artery with renal graft preservation: a case report. Urology **2012**; 80:1151–1153.

8. Taksin L, Mallick S, Frachet O, et al. [Mycotic aneurysm and renal transplant. A case report]. Prog Urol **2009**; 19:149–152.

9. Berbari EF, Kanj SS, Kowalski TJ, et al. 2015 Infectious Diseases Society of America (IDSA) Clinical Practice Guidelines for the Diagnosis and Treatment of Native Vertebral Osteomyelitis in Adults. Clin Infect Dis **2015**; 61:e26-46.

10. Mai H, Champion L, Ouali N, et al. Candida albicans arteritis transmitted by conservative liquid after renal transplantation: a report of four cases and review of the literature. Transplantation **2006**; 82:1163–1167.

11. Baccarani U, Risaliti A, Adani GL, et al. Arterial rupture as the result of fungal arteritis after renal transplantation. Transplantation **2003**; 76:266–269.

12. Battaglia M, Ditonno P, Fiore T, De Ceglie G, Regina G, Selvaggi FP. True mycotic arteritis by Candida albicans in 2 kidney transplant recipients from the same donor. J Urol **2000**; 163:1236–1237.

13. Potti A, Danielson B, Sen K. ‘True’ mycotic aneurysm of a renal artery allograft. Am J Kidney Dis **1998**; 31:E3.

14. Horn A, Summers KL, Fuell W, et al. Salvage of a kidney transplant with a mycotic extrarenal pseudoaneursym using donor vessels. Annals of Vascular Surgery - Brief Reports and Innovations **2024**; 4:100314.

15. Albano L, Bretagne S, Mamzer-Bruneel M-F, et al. Evidence that graft-site candidiasis after kidney transplantation is acquired during organ recovery: a multicenter study in France. Clin Infect Dis **2009**; 48:194–202.

16. Maio R, do Carmo G, do Vale JM, da Gama AD. [Mycotic rupture of the reno-external iliac artery anastomosi following kidney transplantation]. Rev Port Cir Cardiotorac Vasc **2004**; 11:47–50.

17. Gari-Toussaint M, Ngoc LH, Gigante M, et al. [Kidney transplant and Candida albicans arteritis. The importance of analysing the transplant conservation liquid]. Presse Med **2004**; 33:866–868.

18. Dębska-Ślizień A, Chrobak Ł, Bzoma B, et al. Candida arteritis in kidney transplant recipients: case report and review of the literature. Transpl Infect Dis **2015**; 17:449–455.

19. Arterial Mycotic Aneurysm Rupture of Two Kidney Cadaveric Renal Transplantation Recipients Secondary to Enterococci and Candida. Available at: http://turkjnephrol.org/en/arterial-mycotic-aneurysm-rupture-of-two-kidney-cadaveric-renal-transplantation-recipients-secondary-to-enterococci-and-candida-136087. Accessed 19 March 2025.

20. Laouad I, Buchler M, Noel C, et al. Renal artery aneurysm secondary to Candida albicans in four kidney allograft recipients. Transplant Proc **2005**; 37:2834–2836.

21. Rodrigues BF, Natário AS, Vizinho RS, et al. Candida species contamination of preservation fluid-outcome of renal transplantation in 6 patients. Transplant Proc **2013**; 45:2215–2219.

22. Lin Y-H, Liao C-H, Jiang B-J, Chen T-H. Early renal arterial rupture and arterial pseudoaneurysm in graft kidneys from the same deceased donor. Tzu Chi Med J **2018**; 30:250–254.

23. Egbuna O, Pavlakis M, Stillman IE. Acute crescentic glomerulonephritis in a renal allograft: an unusual complication of fungal infection. Am J Kidney Dis **2007**; 50:468–470.

24. El-Bandar N, Kroy DC, Fuller TF, et al. Development of Graft-Site Candidiasis in 3 Solid Organ Transplant Recipients from the Same Donor. Am J Case Rep **2017**; 18:777–781.

25. Jacobs SE, Kirou KA, Seshan SV, Walsh TJ, Hartono C. The Case | Knee pain and allograft dysfunction in a kidney transplant recipient. Kidney Int **2020**; 97:429–430.

26. Praveen G, Khan MF, Augustine R, Siddini V, Vankalakunti M, Ballal HS. Silence before the Storm – Mycotic Aneurysm in Posttransplant Recipient. Indian Journal of Transplantation **2025**; 19:74.

27. Stern S, Bezinover D, Rath P-M, Paul A, Saner FH. Candida Contamination in Kidney and Liver Organ Preservation Solution: Does It Matter? J Clin Med **2021**; 10:2022.

28. Bracale UM, Carbone F, del Guercio L, et al. External iliac artery pseudoaneurysm complicating renal transplantation. Interact Cardiovasc Thorac Surg **2009**; 8:654–660.

29. Calviño J, Romero R, Pintos E, et al. Renal artery rupture secondary to pretransplantation Candida contamination of the graft in two different recipients. Am J Kidney Dis **1999**; 33:E3.

30. Zavos G, Pappas P, Kakisis JD, et al. Endovascular repair as first-choice treatment of iliac pseudoaneurysms following renal transplantation. Transplant Proc **2005**; 37:4300–4302.

31. Madhav D, Kumar P, Mohan C, et al. Candida-associated pseudo-aneurysm of the transplant renal artery presenting as malignant hypertension and managed successfully without nephrectomy. Saudi J Kidney Dis Transpl **2015**; 26:1000–1005.
